# Medial‐pivot total knee arthroplasty demonstrates more constrained mid‐flexion gait kinematics than posterior‐stabilised total knee arthroplasty under standardised soft‐tissue balancing: A randomised controlled trial with a healthy reference cohort

**DOI:** 10.1002/jeo2.70752

**Published:** 2026-05-19

**Authors:** Eiichi Nakamura, Nobukazu Okamoto, Tetsuro Masuda, Satoshi Hisanaga, Masaki Yugami, Yasunari Oniki, Takeshi Miyamoto

**Affiliations:** ^1^ Department of Orthopedic Surgery Kumamoto Kaiseikai Hospital Kumamoto Japan; ^2^ Department of Orthopedic Surgery Kumamoto University Hospital Kumamoto Japan

**Keywords:** gait kinematics, medial‐pivot, randomised controlled trial, soft‐tissue balancing, total knee arthroplasty

## Abstract

**Purpose:**

Most contemporary total knee arthroplasty (TKA) designs aim to replicate native kinematics, yet the independent contribution of implant geometry to dynamic stability remains unclear when soft‐tissue balance is standardised. We compared medial‐pivot (MP) and posterior‐stabilised (PS) TKA implanted under a uniform medial‐tight balancing protocol and contrasted both with healthy knees.

**Methods:**

Thirty‐six patients with medial osteoarthritis were randomised to MP (EVOLUTION®) or PS (Persona®) TKA. All procedures used a medial parapatellar approach, modified measured resection and calibrated medial‐tight balancing. At 1 year, knee injury and osteoarthritis outcome sore (KOOS) and knee society score (KSS) were recorded and three‐dimensional gait kinematics were assessed. Femoral anterior‐posterior translation (FAPT), femoral medial‐lateral translation (FMLT) and tibial rotation angle (TRA) were quantified using a projection‐based algorithm. A healthy reference cohort of 25 young adults (50 knees) completed the identical gait protocol.

**Results:**

KOOS and KSS improved significantly in both TKA groups with no between‐group differences. Maximum stance‐phase FAPT (% body height) differed significantly among groups (analysis of variance [ANOVA] *p* < 0.01), with smaller excursions in MP TKA (0.8 ± 0.6) than PS TKA (1.9 ± 1.0) and healthy knees (1.9 ± 0.8). Maximum FMLT (% body height) also differed (ANOVA *p* < 0.01); MP TKA demonstrated smaller translations (0.9 ± 0.5) than PS TKA (2.4 ± 1.1), while PS TKA exhibited greater translations than healthy knees (1.3 ± 0.7). Maximum internal TRA (degrees) differed significantly (ANOVA *p* < 0.01); MP TKA showed greater internal rotation (4.1 ± 2.2) than PS TKA (2.8 ± 1.7), whereas both were reduced relative to healthy knees (12.0 ± 4.5).

**Conclusion:**

With identical medial‐tight balancing, implant geometry independently influenced gait kinematics. MP TKA exhibited the most constrained translational behaviour, whereas both TKA designs showed reduced tibial rotation relative to healthy knees despite similar clinical outcomes. These findings highlight the biomechanical trade‐off between reproducing native kinematics and adopting implant‐guided stability.

**Level of Evidence:**

Level I, randomised controlled trial.

**Abbreviations**
AP: anterior–posterior
BMI: body mass index
CPAK: coronal plane alignment of the knee
FAPT: femoral anterior–posterior translation
FMLT: femoral medial–lateral translation
FTA: femorotibial angle
IRB: Institutional Review Board
KOOS: knee injury and osteoarthritis outcome score
KSS: knee society score
ML: medial–lateral
MP: medial‐pivot
OA: osteoarthritis
PS: posterior‐stabilised
RCT: randomised controlled trial
ROM: range of motion
SD: standard deviation
TKA: total knee arthroplasty
TRA: tibial rotation angle
UMIN: University Hospital Medical Information Network

## INTRODUCTION

Total knee arthroplasty (TKA) reliably relieves pain and improves function in patients with end‐stage knee osteoarthritis. However, a substantial proportion of patients continue to report dissatisfaction or a persistent sense of instability despite otherwise acceptable surgical outcomes [10, 12, 16, 21]. These complaints have been partly attributed to nonphysiologic knee kinematics after TKA [18, 21, 28, 32], leading implant designers to pursue constructs that more closely reproduce native knee motion.

Medial‐pivot (MP) and posterior‐stabilised (PS) designs represent two distinct approaches towards this goal. The MP concept aims to replicate the asymmetric stability of the native knee by providing a highly constrained medial compartment and a more mobile lateral compartment [1, 8], whereas PS designs rely on a postcam mechanism to guide femoral rollback, particularly at higher flexion angles [20, 34, 43]. Although both designs seek to restore stable and natural movement, prior gait studies have yielded inconsistent results regarding which design better reproduces physiological knee kinematics [4, 30, 36]. Moreover, those studies did not address the variability in soft‐tissue balance, a key determinant of postoperative motion, which has limited the ability to isolate the independent effect of implant geometry in previous research.

To overcome this important confounding factor, the present randomised controlled trial (RCT) compared MP and PS TKA implanted using a standardised medial‐tight balancing protocol to isolate the pure influence of implant geometry on gait behaviour. This approach builds on a previously published RCT, which demonstrated that MP and PS designs exhibit distinct kinematic patterns during demanding weight‐bearing activities such as step‐up and lunge tasks, despite equivalent clinical outcomes when medial soft‐tissue balance is standardised [31]. However, whether these implant‐dependent differences persist during level walking—a fundamental daily activity governed by different neuromuscular and biomechanical demands—remains unclear.

Recent advances in TKA have also focused on alignment strategies, including kinematic alignment and its modifications, such as restricted and functional alignment, which aim to better reproduce native knee phenotype and soft‐tissue behaviour [29, 40]. Because TKA designs are fundamentally grounded in the pursuit of ‘normal‐like’ knee motion [3, 5, 14], it is essential to contextualise postoperative gait kinematics against a normative reference [2]. Therefore, we additionally evaluated a cohort of young healthy adults who underwent the same gait analysis protocol to provide a benchmark for physiologic knee motion during walking. We hypothesised that (1) MP TKA would exhibit more constrained anterior‐posterior and medial‐lateral femoral motion than PS TKA when implanted under identical medial‐tight soft‐tissue conditions, and (2) both TKA designs would deviate from normal knee kinematics during gait, with MP demonstrating a more stabilising—possibly over‐constrained—motion pattern relative to healthy knees. Through this design, we aimed to compare predefined kinematic metrics during gait between MP and PS TKA under standardised medial‐tight balancing, and to contextualise these metrics against healthy reference data.

## MATERIALS AND METHODS

### Study design and participants

This investigation is a prespecified secondary analysis of a prospective, single‐centre, RCT registered prior to enrolment (UMIN‐CTR000033950). Outcomes from this randomised cohort during step‐up and lunge activities have been reported previously [31]; the present manuscript focuses specifically on level‐walking gait kinematics and includes a healthy reference cohort assessed with an identical protocol to contextualise postoperative motion. Patient inclusion and exclusion criteria were the same as those described previously. Briefly, eligible participants had symptomatic medial osteoarthritis scheduled for primary TKA. Patients were excluded if they had inflammatory arthritis, prior knee surgery, concurrent hip or ankle osteoarthritis, preexisting contralateral TKA, cognitive or neurological gait impairment or inability to complete gait testing. Detailed eligibility criteria and procedures for randomisation and allocation concealment are provided in the earlier report. A total of 40 patients were randomised in a 1:1 ratio to MP TKA or PS TKA. The study was approved by the Institutional Review Board of Kumamoto University Hospital, and written informed consent was obtained from all participants.

### Surgical procedure and balancing protocol

All surgeries were performed by two experienced surgeons (E.N. and N.O.) using a medial parapatellar approach and a modified measured resection technique. Detailed descriptions of the surgical workflow and balancing methodology have been previously published [31]. In brief, following bone resection and necessary soft‐tissue releases, a trial femoral component was implanted and gap assessment was performed using an Offset Repo Tensor (Zimmer) placed on the prepared tibial surface. A distraction force of 40 lbs (approximately 18 kg) was applied with a calibrated torque driver to standardise joint tension. The joint centre gap (mm) and varus ligament balance (°) were recorded at six predefined flexion positions (0°, 30°, 60°, 90°, 120° and maximum flexion). Intraoperative balancing aimed to minimise gap variability throughout the range of motion. Specifically, joint centre gap variation was maintained below 5 mm, while varus ligament balance was adjusted to remain between 2° and 5°, thereby establishing a consistent medial‐tight soft‐tissue condition. Patellar resurfacing was not performed.

### Healthy reference group

To provide normative reference data, a separate cohort of healthy young adults (22–29 years) with no history of knee symptoms, injury or surgery was recruited. These participants were not part of the randomised trial and did not undergo TKA. They completed the same gait analysis protocol as the TKA groups to allow comparison of postoperative TKA kinematics with normal knee motion. A total of 25 individuals (50 knees) were included.

### Gait analysis

Gait analysis was performed at 1 year postoperatively for all TKA participants and during a single session for healthy volunteers. A three‐dimensional motion capture system (Locus 3D MA‐3000, Anima Corp.) equipped with eight infrared cameras (sampling frequency: 100 Hz) was used to track the motion of the femur, tibia and rearfoot segments. Eight‐millimetre reflective markers were placed on anatomical landmarks, including the acromion, anterior superior iliac spine, greater trochanter, anterior mid‐thigh, medial and lateral femoral condyles, anterior mid‐crus, medial and lateral malleoli, the head of the fifth metatarsal and the sacral midpoint. Segmental coordinate systems were defined for the femur, tibia and rearfoot.

The femoral long axis was defined as the line connecting the hip and knee joint centres. The hip joint centre was estimated using the anterior superior iliac spine and greater trochanter landmarks, whereas the knee centre was defined as the midpoint between the medial and lateral femoral condyles. The tibial axis was defined by the line connecting the ankle and knee centres, with the ankle centre identified as the midpoint between the medial and lateral malleoli. To quantify tibial rotation more accurately, the femoral plane was constructed using the greater trochanter and bilateral femoral condyles, while the tibial plane was defined using the knee joint centre and bilateral malleoli.

Ground reaction forces were recorded synchronously using two force plates (MG‐100, Anima; 100 Hz) to identify heel‐strike and toe‐off events. Raw force‐plate data were filtered using a fourth‐order Butterworth filter [8] with a cutoff frequency of 20 Hz [42]. A static calibration trial in a relaxed standing posture was used to define anatomical coordinate systems and joint centres.

Participants walked at a self‐selected comfortable speed along an 8‐m walkway. After three familiarisation passes, five successful gait trials were collected for analysis. Kinematic waveforms were time‐normalised to 100% of the gait cycle. The stance phase was defined as 0%–60% of the gait cycle. For each kinematic variable, the maximum translation or rotation was defined as the maximum absolute value observed during the stance phase, representing the greatest excursion experienced under load. These maximum stance‐phase values were used for subsequent statistical analyses. We used these maximum absolute stance‐phase excursions as summary metrics to compare the greatest under‐load translations/rotations between groups, but the waveform plots are provided for descriptive visualisation and were not subjected to time‐series inferential testing. Three primary parameters were evaluated: (1) femoral anterior‐posterior translation (FAPT), (2) femoral medial‐lateral translation (FMLT) and (3) tibial rotation angle (TRA).

A tibial axis line was extended proximally from the ankle marker, and the perpendicular projection of the knee centre onto this line defined a virtual patellar reference point. The knee‐reference point distance in the sagittal and frontal planes represented FAPT and FMLT, respectively. TRA quantified the relative orientation between the femoral and tibial planes.

### Clinical outcome measures

Clinical assessments were performed preoperatively and at 1 year using the knee injury and osteoarthritis outcome score (KOOS) and knee society score (KSS). Examiners were blinded to implant allocation.

### Statistical analysis

Statistical analyses were performed using commercially available software. Analyses of the randomised comparison were conducted on a knee‐by‐knee basis, as each participant contributed a single operated knee. Continuous variables were assessed for normality and are presented as mean ± standard deviation. Baseline characteristics were compared between groups using the Mann‐Whitney *U*‐test or chi‐square test as appropriate.

For gait kinematic variables included FAPT, FMLT and TRA, each maximum absolute value observed during the stance phase (0%–60% of the gait cycle) was extracted and used for statistical comparisons. Between‐group differences between MP and PS TKA were assessed using independent‐samples *t*‐tests or nonparametric equivalents when assumptions were violated. For exploratory comparisons including the healthy reference cohort, one‐way analysis of variance with Tukey post hoc testing was performed. A two‐sided *p*‐value < 0.05 was considered statistically significant. Sample size determination was performed a priori for the parent RCT; no additional power analysis was conducted for the present gait‐specific outcomes. This gait analysis represents a prespecified secondary analysis of the parent RCT. The original sample size calculation was not based on gait kinematic outcomes; therefore, both statistically significant and nonsignificant findings should be interpreted cautiously.

### Ethical aspects

This study was approved by the Institutional Review Board of Kumamoto University Hospital (Approval No. 2376). The study protocol was registered with the University Hospital Medical Information Network Clinical Trials Registry (UMIN‐CTR 000033950). All procedures were conducted in accordance with the Declaration of Helsinki. Written informed consent was obtained from all participants.

## RESULTS

### Patient characteristics

**Figure 1:**
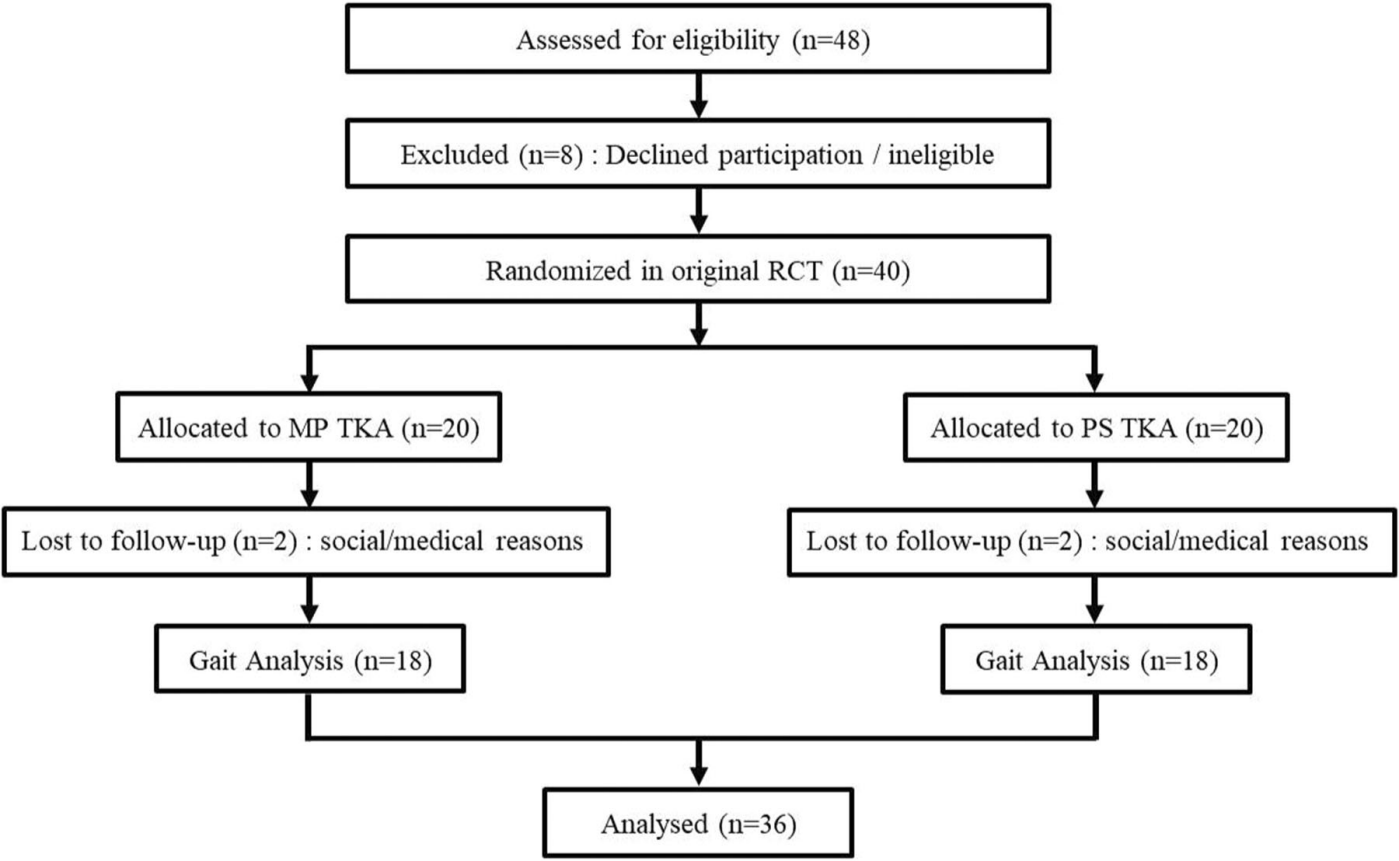
CONSORT flow diagram of participant inclusion for gait analysis. Flow diagram summarising participant enrolment, randomisation and inclusion in the gait analysis. Thirty‐six participants (18 MP TKA and 18 PS TKA) completed the 1‐year gait assessment and were included in the present analysis. MP TKA, medial‐pivot total knee arthroplasty; PS TKA, posterior‐stabilised total knee arthroplasty; RCT, randomised controlled trial.

The present study represents an analysis of gait data derived from a previously reported RCT cohort [31]. Of the 40 patients initially randomised (20 MP, 20 PS), four did not complete the 1‐year gait assessment (two MP, two PS) because of medical or social reasons. Thus, gait analysis was performed in 36 patients, corresponding to 36 operated knees (MP: 18 patients/18 knees; PS: 18 patients/18 knees). A flow diagram summarising participant inclusion is shown in Figure 1. Baseline demographic characteristics did not differ significantly between groups (Table 1). A separate cohort of 25 healthy young adults (50 knees) served as a normative reference for gait comparison, and their characteristics are presented in Table 2.

**Table 1:** Baseline characteristics of patients included in gait analysis.

		MP group	PS group	*p‐*value
Knees		18	18	‐
Age at surgery (years)		76 ± 6	78 ± 6	0.125
Gender (male/female)		8/10	5/13	0.297
Height (cm)		154.8 ± 8.4	154.2 ± 7.8	0.877
BMI (kg/m^2^)		26.2 ± 2.9	25.6 ± 3.9	0.863
ROM (⁰)				
Extension		6 ± 6	4 ± 5	0.999
Flexion		124 ± 6	125 ± 6	0.578
KOOS	Global	43.8 ± 11.2	46.2 ± 18.3	0.650
KSS	Symptom	9.2 ± 5.6	6.9 ± 4.4	0.204

*Note*: Data are presented as mean ± standard deviation.

Abbreviations: BMI, body mass index; KOOS, knee injury and osteoarthritis outcome scores; KSS, knee society scores; MP group, medial‐pivot total knee arthroplasty; PS group, posterior‐stabilised total knee arthroplasty; ROM, range of motion.

**Table 2:** Demographic characteristics of the healthy reference cohort.

	Healthy group
Knees	50
Age (years)	24 ± 2
Gender (male/female)	12/13
Height (cm)	167.1 ± 8.0
BMI (kg/m^2^)	21.7 ± 2.4
ROM (⁰)	
Extension	2 ± 2
Flexion	138 ± 6

*Note*: Data are presented as mean ± standard deviation.

Abbreviations: BMI, body mass index; ROM, range of motion.

### Radiographic and clinical outcomes

There were no statistically significant differences in the demographic data and clinical scores preoperatively (Table 1). There were also no statistically significant between‐group differences in postoperative limb alignment (femorotibial angle [FTA]) or component positioning (Table 3). At 1 year, knee extension was 1° ± 2° in both groups, and knee flexion was 121° ± 6° (MP) versus 125° ± 7° (PS), with no significant difference. Both groups demonstrated significant improvements in all KOOS subscales and in KSS clinical and functional scores from baseline to 1 year; however, no between‐group differences were detected for any clinical outcome measure (Table 3).

**Table 3:** Radiographic alignment, implant positioning and clinical outcomes in MP and PS TKA.

		MP group	PS group	*p‐*value
FTA (°)	Preop.	183 ± 5	181 ± 5	0.329
	Postop.	175 ± 2	176 ± 2	0.176
Component position (°)	*α*	95.7 ± 1.8	95.6 ± 1.4	0.781
	*β*	89.2 ± 1.1	88.9 ± 1.9	0.561
	*γ*	1.2 ± 0.9	1.2 ± 0.5	0.981
	*δ*	86.7 ± 2.6	87.4 ± 1.4	0.342
Postop. KOOS	Symptom	81.0 ± 11.4	80.9 ± 13.3	0.986
	pain	82.1 ± 12.4	86.9 ± 15.4	0.325
	ADL	79.5 ± 11.9	83.9 ± 13.0	0.303
	sports/recreation	38.6 ± 28.6	44.7 ± 24.5	0.503
	QOL	53.8 ± 23.5	67.3 ± 20.8	0.082
	global	74.0 ± 12.5	78.0 ± 13.2	0.366
Postop. KSS	Symptom	18.6 ± 3.6	20.0 ± 4.8	0.327
	Satisfaction	24.6 ± 6.4	28.8 ± 6.8	0.103
	Expectation	10.3 ± 3.1	10.5 ± 3.2	0.858
	Gait	21.3 ± 7.9	21.4 ± 5.5	0.954
	Activity	22.0 ± 5.2	24.4 ± 4.7	0.168
	Activity2	12.1 ± 6.3	13.1 ± 4.6	0.594
	Exercise	9.0 ± 3.5	11.3 ± 4.3	0.108

*Note*: Data are presented as mean ± standard deviation.

Abbreviations: ADL, activities of daily living; FTA, femorotibial angle; KOOS, knee injury and osteoarthritis outcome scores; KSS, knee society scores; MP group, medial‐pivot total knee arthroplasty; PS group, posterior‐stabilised total knee arthroplasty; QOL, quality of life; TKA, total knee arthroplasty.

### Gait kinematics

**Figure 2:**
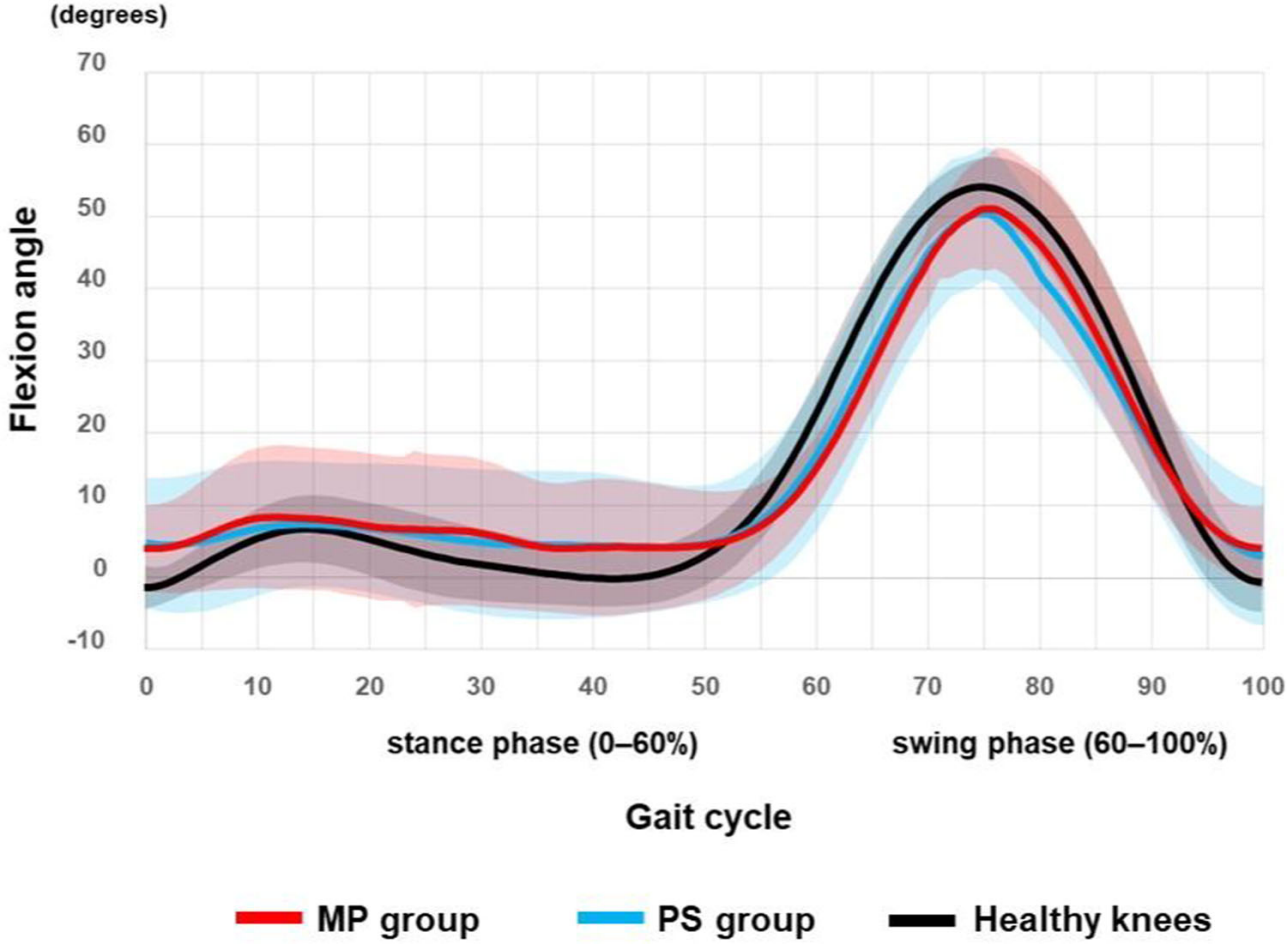
Knee flexion‐extension angle during gait. Red: medial‐pivot (MP) TKA; blue: posterior‐stabilised (PS) TKA; black: healthy knees. Mean knee flexion‐extension angle throughout the gait cycle is shown for MP TKA, PS TKA and healthy knees. The gait cycle was time‐normalised to 100%, with the stance phase defined as 0%–60% and the swing phase as 60%–100%. Solid lines represent group mean waveforms, and shaded areas indicate ±1 standard deviation. Waveforms are presented for descriptive purposes only and do not imply statistically verified differences across the gait cycle. TKA, total knee arthroplasty.

**Figure 3:**
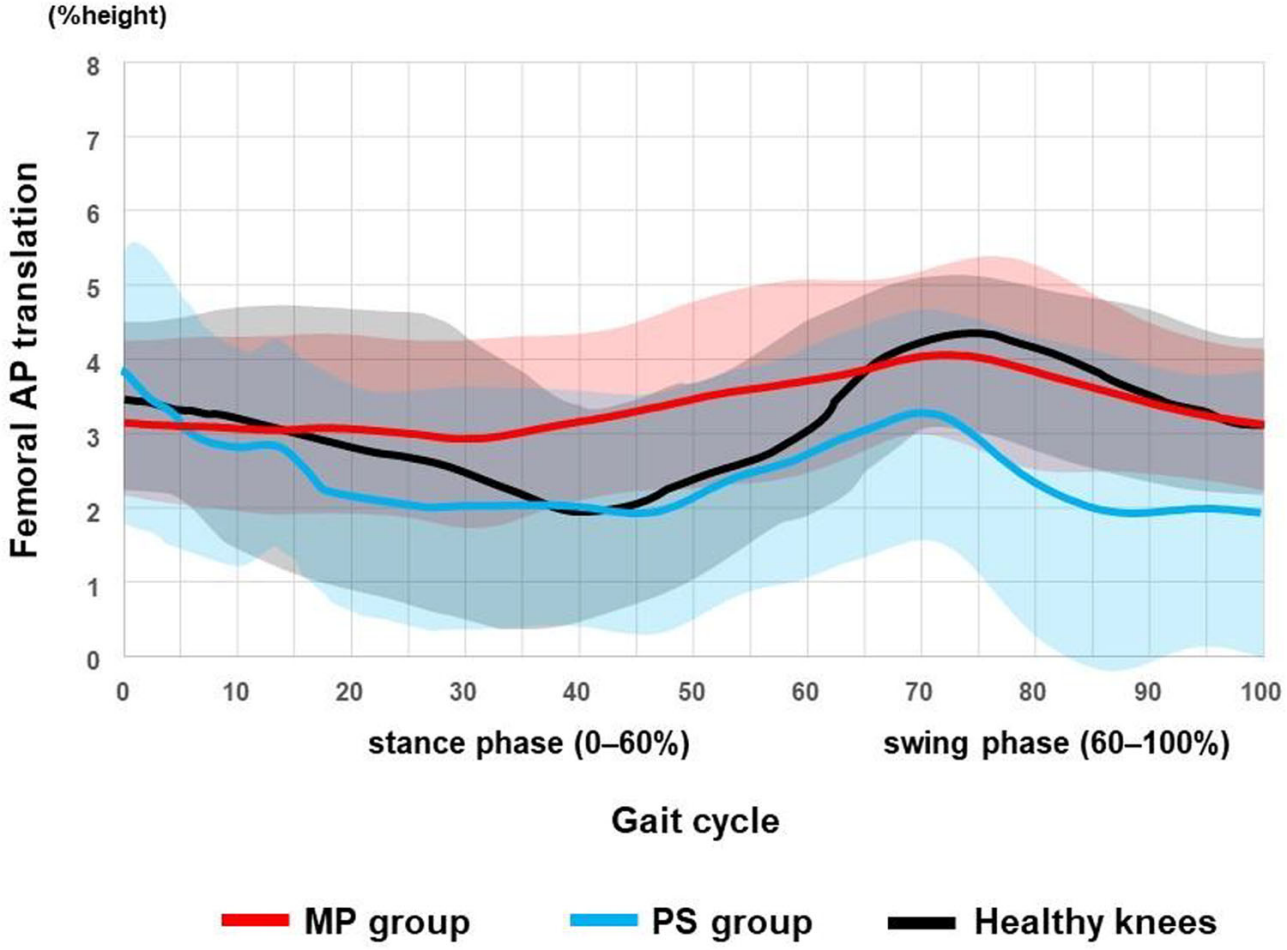
Femoral anterior‐posterior translation (FAPT) during gait. Red: medial‐pivot (MP) TKA; blue: posterior‐stabilised (PS) TKA; black: healthy knees. Mean FAPT throughout the gait cycle is shown for MP TKA, PS TKA and healthy knees. The gait cycle was time‐normalised to 100%, with the stance phase defined as 0%–60%. Solid lines represent group mean waveforms and shaded areas indicate ±1 standard deviation. Waveforms are presented for descriptive purposes only and do not imply statistically verified differences across the gait cycle. TKA, total knee arthroplasty.

**Figure 4:**
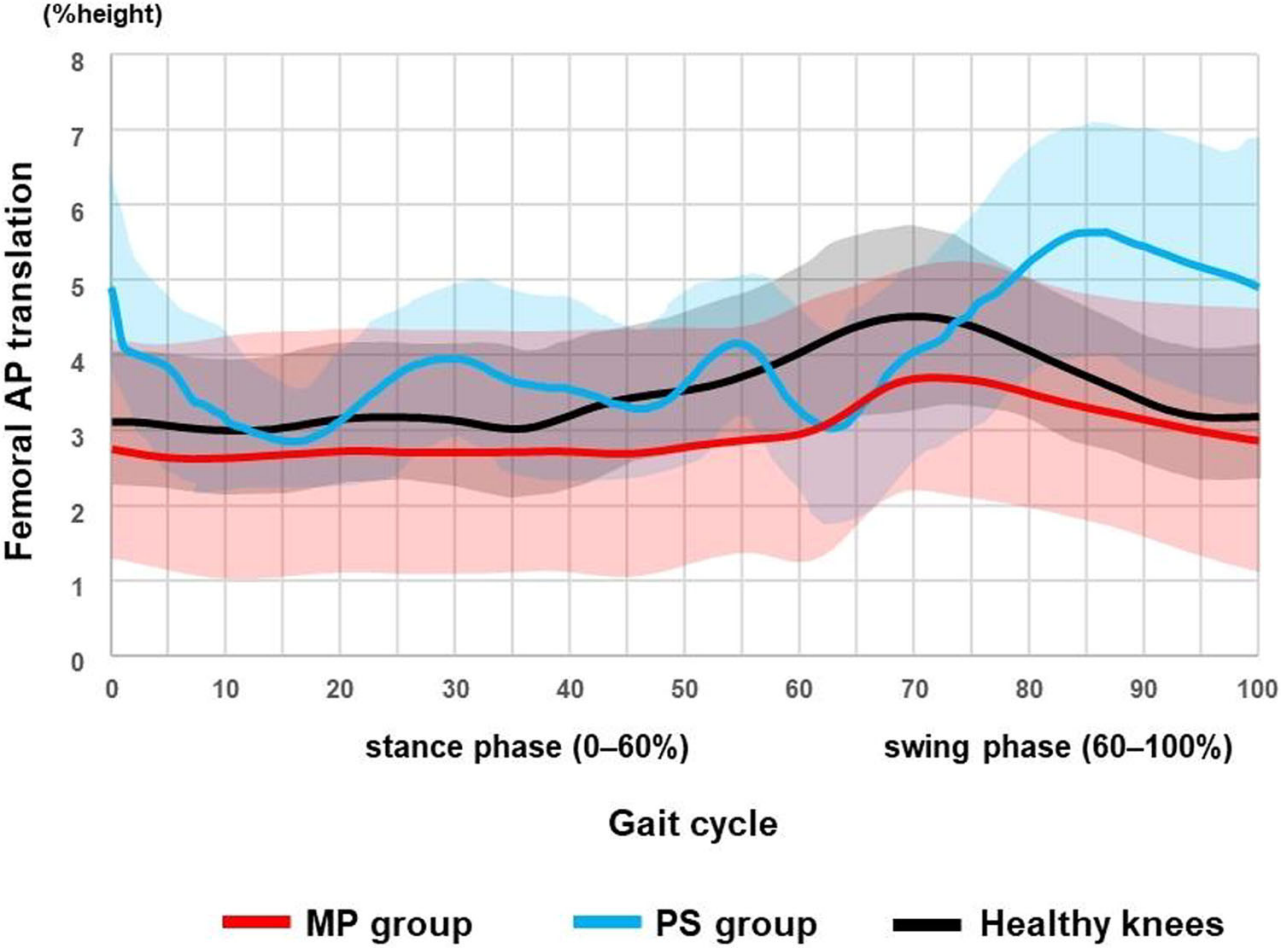
Femoral medial‐lateral translation (FMLT) during gait. Red: medial‐pivot (MP) TKA; blue: posterior‐stabilised (PS) TKA; black: healthy knees. Mean FMLT throughout the gait cycle is shown for MP TKA, PS TKA and healthy knees. The gait cycle was time‐normalised to 100%, with the stance phase defined as 0%–60%. Solid lines represent group mean waveforms, and shaded areas indicate ±1 standard deviation. Waveforms are presented for descriptive purposes only and do not imply statistically verified differences across the gait cycle. TKA, total knee arthroplasty.

**Figure 5:**
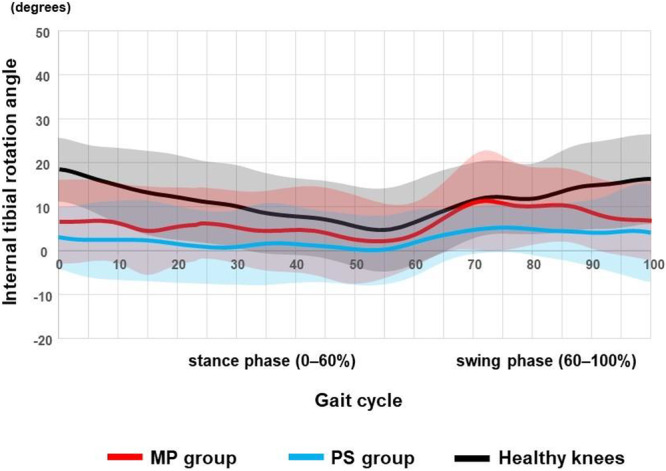
Internal tibial rotation angle (TRA) during gait. Red: medial‐pivot (MP) TKA; blue: posterior‐stabilised (PS) TKA; black: healthy knees. Mean TRA throughout the gait cycle is shown for MP TKA, PS TKA and healthy knees. The gait cycle was time‐normalised to 100%, with the stance phase defined as 0%–60%. Solid lines represent group mean waveforms, and shaded areas indicate ±1 standard deviation. Waveforms are presented for descriptive purposes only and do not imply statistically verified differences across the gait cycle. TKA, total knee arthroplasty.

Waveform plots (Figures 2, 3, 4, 5) are descriptive visualisations only; statistical comparisons were performed exclusively on predefined maximum stance‐phase values.

### Knee flexion‐extension angle

Knee flexion‐extension patterns during gait appeared similar among MP, PS and healthy knees (Figure 2). No significant between‐group differences were observed in the maximum stance‐phase knee flexion angle or in the timing of flexion‐extension transitions. These findings indicate that overall sagittal‐plane gait behaviour was comparable across groups, suggesting that subsequent differences in AP/ML translations and tibial rotation were not attributable to alterations in global gait patterns.

### FAPT

Descriptively, FAPT during stance appeared smaller in MP knees than in PS knees (Figure 3). Consistent with this observation, the maximum absolute FAPT during stance was significantly smaller in the MP group than in the PS group and healthy knees (Table 4), indicating smaller maximum stance‐phase anterior–posterior excursions in MP TKA.

**Table 4:** Maximum stance‐phase translations and rotations in medial‐pivot and posterior‐stabilised TKA, and healthy knees.

	MP group	PS group	Healthy knees	Overall *p*‐value	Post hoc
FAPT(% body height)	0.8 ± 0.6**, *^#^*	1.9 ± 1.0	1.9 ± 0.8	<0.01	MP < PS; MP < healthy
FMLT (% body height)	0.9 ± 0.5**	2.4 ± 1.1	1.3 ± 0.7	<0.01	MP < PS; PS > healthy
Internal TRA (degrees)	4.1 ± 2.2*, *^#^*	2.8 ± 1.7#	12.0 ± 4.5	<0.01	MP > PS; MP, PS < healthy

*Note*: Values are presented as mean ± standard deviation. Internal tibial rotation values in healthy knees reflect stance‐phase excursion during walking. Abbreviations: FAPT, femoral anterior‐posterior translation; FMLT, femoral medial‐lateral translation; MP group, medial‐pivot total knee arthroplasty; PS group, posterior‐stabilised total knee arthroplasty; TKA, total knee arthroplasty; TRA, tibial rotation angle.

* *p* < 0.05

** *p* < 0.01: MP group versus PS group.

^#^ *p* < 0.01: MP group, PS group versus Healthy knees.

### FMLT

Descriptively, femoral medial–lateral translation appeared smaller in MP knees (Figure 4). Quantitatively, the maximum absolute stance‐phase femoral medial–lateral translation was significantly smaller in the MP group than in the PS group, while PS knees demonstrated greater medial‐lateral translation than healthy knees (Table 4).

### TRA

Tibial rotation patterns during stance appeared reduced in both TKA groups compared with healthy knees (Figure 5). The maximum absolute internal tibial rotation during stance differed significantly among groups (Table 4), with MP TKA demonstrating greater internal rotation than PS TKA, whereas both TKA designs exhibited reduced internal rotation compared with healthy knees.

### **Maximum stance‐phase translations and rotations** (Table 4)

Maximum stance‐phase translations and rotations differed significantly among MP TKA, PS TKA and healthy knees (Table 4). The maximum absolute FAPT during stance was significantly smaller in the MP group than in the PS group and healthy knees. The maximum absolute FMLT during stance also showed overall group differences; post hoc testing demonstrated smaller FMLT in MP than PS, and greater FMLT in PS than healthy knees. The maximum absolute internal TRA differed among groups as well: MP showed greater internal rotation than PS, whereas both TKA groups exhibited reduced internal rotation compared with healthy knees. Because statistical comparisons were defined a priori on maximum stance‐phase values, these findings should be interpreted as differences in peak excursion magnitude rather than time‐dependent differences across the entire gait cycle.

## DISCUSSION

The most important finding of this study is that under an identical medial‐tight balancing protocol, MP TKA exhibited significantly smaller maximum femoral anterior‐posterior and medial‐lateral translations during stance‐phase walking compared with PS TKA, indicating smaller maximum stance‐phase femoral translations during walking. This RCT demonstrates that, when soft‐tissue balance is rigorously standardised, implant geometry plays a substantial and directionally consistent role in shaping postoperative gait kinematics after TKA.

Consistent with prior literature [4, 36, 46], both TKA designs deviated from healthy knee kinematics. In the present study, the healthy cohort primarily served as a normative reference obtained using an identical measurement and processing pipeline. Relative to the healthy reference values, MP TKA demonstrated smaller maximum stance‐phase excursions. This observation should be interpreted as a difference in peak excursion magnitude rather than as a restoration or loss of physiological behaviour [17, 36].

These observations directly challenge a long‐standing assumption in TKA design—that reproducing native knee kinematics is inherently desirable [11, 13]. Although many contemporary implants have been developed based on this principle, the extent to which such designs actually achieve physiological in vivo motion remains uncertain. Knee kinematics are highly task‐dependent, underscoring the importance of evaluating multiple functional activities rather than relying on a single movement pattern [13, 41]. While there is general agreement that, during loaded knee flexion, the healthy knee exhibits a characteristic MP pattern characterised by relative medial stability and lateral femoral rollback [9, 20, 34], the kinematic patterns observed during gait remain controversial. Prior investigations have reported MP, lateral‐pivot and mixed patterns during walking, with no clear consensus across studies [26, 27, 30, 35].

Importantly, the present gait analysis demonstrates that even when soft‐tissue balance—often cited as a major determinant of postoperative kinematic variability—is carefully controlled, neither MP nor PS designs reproduce the rotational and translational patterns observed in healthy knees during walking. Instead, each implant follows a distinct kinematic trajectory characteristic of its intrinsic geometry: MP TKA exhibits greater restraint in anterior‐posterior translation and reduced rotational excursion, whereas PS TKA allows greater medial‐lateral translation with similarly reduced rotational amplitudes compared with normative values. Given the comparable knee flexion‐extension waveforms across the gait cycle among MP TKA, PS TKA and healthy knees, the magnitude and direction of the deviations observed in both TKA designs are unlikely to be attributable solely to age‐related differences [38], suggesting a design‐specific influence on knee kinematics.

Despite these clear biomechanical differences, clinical outcomes at 1 year—as assessed by KOOS and KSS—did not differ between MP and PS designs. This finding is consistent with previous systematic reviews and meta‐analyses demonstrating no consistent superiority of MP TKA over PS TKA in terms of patient satisfaction or perceived function [6, 22, 33, 44]. In addition, variations in surgical technique, such as PCL preservation or sacrifice in medial pivot TKA, have been reported to have limited impact on clinical outcomes [24]. The dissociation between dynamic kinematic measures and short‐term patient‐reported outcomes highlights a persistent challenge in TKA research: biomechanical advantages observed under controlled experimental conditions do not necessarily translate into early clinical benefit [23, 33, 44].

The findings of the present study provide insight into a key contemporary issue in TKA: whether efforts should focus on pursuing closer reproduction of native knee kinematics [3, 5], or whether implant‐guided motion patterns that prioritise stability should be intentionally adopted [7], even at the expense of physiological fidelity. The present findings should also be interpreted in the context of evolving alignment philosophies, as implant geometry and alignment strategy are likely to interact in determining postoperative knee kinematics. Recent studies have demonstrated that kinematic alignment can restore native knee phenotype in the short term, while variations in coronal alignment classification do not necessarily result in clinically meaningful differences in outcomes following restricted kinematic alignment TKA [28, 40]. In addition, functional alignment has been reported to maintain consistent medial stability and promote a more distinct medial pivot pattern compared with mechanical alignment in MP TKA [19, 25], suggesting that alignment strategy may influence the expression of implant‐specific kinematic characteristics. Furthermore, recent classification systems such as the coronal plane alignment of the knee framework have emphasised the wide variability in native knee phenotypes and the importance of individualised alignment strategies [15]. Taken together, these findings indicate that postoperative knee kinematics are not solely determined by implant design but rather reflect a complex interaction between implant geometry, alignment philosophy and soft‐tissue balance. Although restoration of physiological knee motion has long been considered a fundamental goal of TKA, consistent reproduction of native knee kinematics remains difficult, even with anatomically inspired designs or ligament‐preserving implants [5, 47]. Moreover, excessive pursuit of kinematic fidelity may increase variability in postoperative movement patterns, potentially leading to patient dissatisfaction, including residual instability. Looking ahead, advances in technologies such as robotic‐assisted TKA [37], and compartment pressure sensors [39, 48]. which enable more accurate reproduction of implant positioning and objective assessment of soft‐tissue balance—are expected to play an important role in improving our understanding of optimal alignment strategies and implant design. Implant geometry may influence peak stance‐phase excursions even when identical balancing protocols are applied, and previous studies have suggested that anterior–posterior femoral translation patterns are associated with postoperative functional outcomes such as range of motion [45]. Surgeons may consider implant constraint characteristics alongside intended balance targets to avoid potential over‐constraint or residual laxity. However, we did not compare alternative alignment or balancing strategies, nor did we evaluate long‐term clinical outcomes; therefore, these implications remain exploratory.

This study represents a secondary analysis and was not specifically powered for gait kinematics. Consequently, effect estimates may be imprecise, and the possibility of type I and type II errors should be considered. And, we did not evaluate alternative balancing strategies or establish thresholds linking kinematic excursions to patient‐reported instability or long‐term outcomes. These findings should be interpreted in light of the study limitations discussed below. The main limitations of this study are the relatively small sample size and the short follow‐up period. The relatively small sample size per TKA group may limit the detection of subtle or time‐localised differences in kinematic waveforms and restrict generalisability. Although knee kinematics after TKA change over time, short‐term follow‐up remains valuable for assessing early functional recovery and identifying kinematic patterns potentially associated with instability or implant‐related issues; nevertheless, larger cohorts with longer follow‐up are needed. Second, although soft‐tissue balance was carefully standardised, the surgeon was not blinded to the implant type, and related bias cannot be completely excluded. Third, the present analysis was limited to level walking. While our previous work focused on lunge and step‐up tasks under higher functional demands, gait was selected to evaluate dynamic knee stability during the most fundamental daily activity after TKA, providing complementary information under repetitive functional loading. Finally, although a healthy reference cohort was included, the age and body mass index (BMI) differences between patients and controls should be considered when interpreting comparisons with native knee kinematics. These differences reflect inherent demographic variation between young asymptomatic individuals and elderly arthroplasty patients. Age and BMI may influence gait mechanics and, therefore, represent potential sources of bias when evaluating deviations from physiological knee motion. However, the healthy cohort was intended to provide a physiological reference rather than a matched control population. Importantly, the principal randomised comparison between MP and PS TKA was conducted within age‐ and BMI‐comparable patient groups, reducing the likelihood that demographic factors substantially influenced the primary between‐implant findings.

## CONCLUSIONS

Under a standardised medial‐tight ligament balancing protocol, MP and PS TKAs demonstrated distinct gait kinematics, indicating that implant geometry independently influences dynamic knee behaviour during walking. Neither design fully reproduced native knee kinematics, although MP TKA exhibited a more constrained motion pattern during stance‐phase gait.

## AUTHOR CONTRIBUTIONS

Eiichi Nakamura designed the study, conducted data interpretation and drafted the manuscript. Nobukazu Okamoto, Tetsuro Masuda and Satoshi Hisanaga contributed to study design, surgeries and data acquisition. Masaki Yugami and Yasunari Oniki contributed to data analysis and interpretation. Takeshi Miyamoto supervised the study and critically revised the manuscript. All authors read and approved the final manuscript.

## CONFLICT OF INTEREST STATEMENT

The authors declare no conflicts of interest.

## ETHICS STATEMENT

This study was approved by the Institutional Review Board of Kumamoto University Hospital (Approval No. 2376). Written informed consent was obtained from all participants prior to enrolment.

## Data Availability

The datasets generated and/or analysed during the current study are available from the corresponding author on reasonable request.
